# Magnitude, components and predictors of metabolic syndrome in Northern Ethiopia: Evidences from regional NCDs STEPS survey, 2016

**DOI:** 10.1371/journal.pone.0253317

**Published:** 2021-06-21

**Authors:** Kiros Fenta Ajemu, Abraham Aregay Desta, Asfawosen Aregay Berhe, Ataklti Gebretsadik Woldegebriel, Nega Mamo Bezabih, Degnesh Negash, Alem Desta Wuneh, Tewolde Wubayehu Woldearegay

**Affiliations:** 1 Tigray Health Research Institute, Mekelle, Tigray, Ethiopia; 2 College of Health Science, Mekelle University, Mekelle, Tigray, Ethiopia; Università degli Studi di Milano, ITALY

## Abstract

**Background:**

Individuals with metabolic syndrome are five times more susceptible to chronic diseases. Assessment of its magnitude, components, and risk factors is essentials to deploy visible interventions needed to avoid further complications. The study aimed to assess magnitude, components, and predictors of metabolic syndrome in Tigray region northern Ethiopia, 2016.

**Methods:**

Data were reviewed from Tigray region NCDs STEPs survey data base between May to June 2016. A total of 1476 adults aged 18–64 years were enrolled for the study. Multi-variable regression analysis was performed to estimate the net effect of size to risk factors associated with metabolic syndrome. Statistical significance was declared at p-value of ≤0.05 at 95% confidence interval (CI) for an adjusted odds ratio (AOR).

**Results:**

The study revealed that unadjusted and adjusted prevalence rate of Metabolic Syndrome (MetS) were (CPR = 33.79%; 95%CI: 31.29%–36.36%) and (APR = 34.2%; 95% CI: 30.31%–38.06%) respectively. The most prevalent MetS component was low HDL concentration (CPR = 70.91%; 95%CI: 68.47%–73.27%) and (APR = 70.61; 95%CI; 67.17–74.05). While; high fasting blood glucose (CPR = 20.01% (95%CI: 18.03–22.12) and (APR = 21.72; 95%CI; 18.41–25.03) was the least ones. Eating vegetables four days a week, (AOR = 3.69, 95%CI; 1.33–10.22), a salt sauce added in the food some times (AOR = 5.06, 95%CI; 2.07–12.34), overweight (AOR = 24.28, 95%CI; 10.08–58.47] and obesity (AOR = 38.81; 12.20–111.04) had strong association with MetS.

**Conclusion:**

The magnitude of metabolic syndrome was found to be close to the national estimate. Community awareness on life style modification based on identified MetS components and risk factors is needed to avoid further complications.

## Introduction

Metabolic syndrome (MetS) has received much attention in recent times. It is a cluster of cardiovascular risk factors in the tune of abdominal obesity, hyperglycemia, dyslipidemia and high blood pressure [1–3]. Globally, adult population having MetS ranged from 20–25%. Individuals with metabolic syndrome are five times more susceptible to chronic diseases [3–6] and becoming an important cause of morbidity and mortality in Africa. Pieces of evidences suggested that contributing factors were rapid demographic transition, changing behaviors, and lifestyles [2–5]. The high MetS prevalence was observed in Africa ranged from 17–25% [7]. In particular; North—Western Nigeria (35.1%), South Africa (21.8%), Morocco (35.4%), and Cameroon (38.9%) [8–11].

However, in Sub-Saharan Africa (SSA) its prevalence will be 59% to 179% in 2030 [2–5]. Even though most of the studies on MetS were conducted in North America, Europe, and Asia [12–14], its impact was high in sub-Saharan African; like Kenya (25.6%), and Tanzania (38%) [15–17].

In Ethiopia the change in lifestyle due to the current rapid economic growth increased the burden of MetS [18] with an overall pooled prevalence of 20.3% [18]. Accordingly, evidence from adult treatment panel (ATP III) and international diabetic federation (IDF) showed prevalence of 12.5% and 17.9% [19–21].

Considering the literature gap on MetS prevalence and risk factors, the study aimed to assess the prevalence, components and predictors of metabolic syndrome. The evidence will use as a preliminary report to estimate the epidemiology of metabolic syndrome that will be used to promote health promotion and prevention activities for life style modification and action towards metabolic syndrome control and management.

## Materials and methods

### Design and setting

The study involved a community based cross-sectional study design. It was conducted in Tigray region Northern Ethiopia located 802KMs from Addis Ababa, the administrative capital city of Ethiopia [22]. Tigray region is the homeland of the Tigrayan, Irob and Kunama peoples. Tigray is also known as Region 1 according to the federal constitution. Its capital and largest city is Mekelle. Tigray norther Ethiopia is the 5th largest by area, the 5th most populous, and the 5th most densely populated of the 10 Regional States. Estimated total population is 7,070,260 [22] The region is further administratively subdivided into seven zones, namely, East, South, South East, Western, Northwestern, Central, and Mekelle which contained the smallest administrative units of 52 districts (34 rural and 18 urban). The study period was between May to June 2016.

### Exclusion criteria

Pregnant women and critically ill patients were excluded from the study.

### Sampling and sample size determination

As this study was part of previously published work [22], further details on the sampling technique, data collection procedure were described there. The study subjects were respondents with age categories between 18–64 years. Data were obtained from the 2016 regional STEPs survey data base (S1 Dataset). Sample size was calculated using single proportion formula; where Z-score Z α /2 = 1.96 at 95% confidence level (CL), a margin of error (d) = 5% (0.05) and assuming MetS prevalence of 18.9% (0.189) [19].


$$\text{n}=\frac{{\left(1.96\right)}^{2}\times 0.189\left(0.811\right)}{{\text{D}}^{2}={\left(0.05\right)}^{2}}$$

n=227

The total sample size was 250 by considering a 10% non-response rate. But we included 1476 respondents that had complete list of variables in the data base.

### Data collection

Data collection was carried out using a standardized questionnaire (S1 File) adopted from WHO (STEPS) instrument with slight modifications. Items had strong internal consistency (α = 0.925) [23]. Initially, the tool was prepared in English and translated into Tigrigna (local language).Three male and five female nurses with college degree and at least five-year clinical experience were recruited as data collectors. They were trained for five days on interview skills, the standard physical measurements following the WHO guideline, and blood test procedures using portable analysers. During the training, the eight data collectors conducted interviews, physical measurements, and blood tests to each two volunteers. The survey procedure was modified according to the feedback of data collectors and volunteers during the training. Two supervisors monitored the quality of the data collection. The time taken for each interview was 15 minutes. The study protocol was reviewed and approved by Mekelle University School of Public Health ethical review board. Permission was also received from Tigray Regional Health Bureau and respective health facilities. Similarly, data collection was conducted if and only if an informed consent was approved from study participant.

### Measurement and classification

After measuring each variable, the classification of MetS risk factors was made based on the cut-off values of diagnosis reference of National Heart, Lung, and Blood Institute (NHLBI) [21, 24–27].

### Operational definitions

#### A waist circumference (WC)

Waist circumference of 35 inches or more for women or 40 for men.

#### A triglyceride level

Triglyceride level of 150 mg/dl or higher or being on medicine to treat high triglycerides.

#### Cholesterol (HDL-C)

Sometimes is called "good" cholesterol with a level of less than 50 mg/dl for women and less than 40 mg/dl for men.

#### Blood pressure (BP)

Blood pressure of 130/85 mmHg or higher or being on medicine to treat high blood pressure.

#### Fasting blood sugar level

Fasting blood sugar was considered normal for less than 100 mg/dL; pre-diabetic if between 100–125 mg/dL; while a fasting blood sugar level of 126 mg/dL or higher was considered as diabetes and a fasting blood sugar level of 100 mg/dL.

#### A salt sauce added in the food

Rarely (2 grams/day); sometimes (< 2 grams/day); always; (>2 grams/day).

#### Physical inactivity

< 600MET-minutes per week.

#### Low fruit and vegetable consumption

< five servings per day.

#### Alcohol

≥4 standard drinks per day for men; ≥3 standard drinks per day for women.

#### Metabolic syndrome

Was considered if at least three metabolic syndromes are present, according to the National Cholesterol Education Program’s Adult Treatment Panel III (NCEP-ATP III).

### Data quality assurance

Data collectors were trained for two days. The survey procedure was modified according to the feedbacks provided during training. Completed questionnaires were checked daily by the supervisor and principal investigator.

### Data processing and analysis

Data were entered and processed using Epi-Info version 7.1.5 (Center for Disease Control and Prevention, USA) and analyzed using SPSS version 21.0 (SPSS Chicago, IL, USA). Descriptive data were presented in tables. The prevalence was described in terms of Crude & Adjusted Prevalence Rate (APR, CPR). The binary logistic regression model was used to see the net effect size. The net effect size was interpreted at P-value ≤ 0.05. Overall fitness of the model was evaluated for each logit function. The final model fitness was checked using Hosmer and Lemeshow test. Model was considered good and fit since p -value was more than 0.05 from the Hosmer-Lemeshow test. Interactions of variables were assessed at p-value < = 0.05 and confounding of variables were assessed by backward and forward elimination and any variable which had > 20% change of coefficient of the parameters between the reduced and full model was considered as confounder. Similarly, collinearity was checked by Variance Inflation Factor (VIF) and If VIF was greater than 10 it was considered as collinear and removed from the model.

## Result

### Socio-demographic characteristics

A total of 1476 respondents were enrolled in the study. Of these, 842 (57%) were females. Almost closer to half (42.7%) were age category between 29–39 years. Majorities (94%) were orthodox Christian (Table 1).

**Table 1:** Frequency distribution of socio-demographic characteristics of respondents in Northern Ethiopia (n = 1476).

Variables	Category	Frequency	
		Number	Percent
Sex	Male	842	57
	Female	634	43
Age in years	18–28	240	16.3
	29–39	630	42.7
	40–50	416	28.2
	51–61	162	11
	62+	228	1.8
Marital status	Currently married	366	24.8
	Never married	962	65.2
	Separated/divorced/widowed	148	10
Education level	≤8 Grade level	101	6.8
	9–12 Grade Level	175	11.9
	>12 Grade level	1200	81.3
Religion	Orthodox	1387	94
	Muslim	50	3.4
	Protestant	39	2.6

### Prevalence and components of metabolic syndrome

The most prevalent MetS component was low HDL concentration CPR = 70.91% (95%CI: 68.47%–73.27%) and APR = 70.61% (95%CI: 67.17%–74.05%). While; high fasting blood glucose CPR = 20.01% (95%CI: 18.03%–22.12%) and APR = 21.72% (95%CI: 18.41–25.03) was the least one (Table 2).

**Table 2:** Prevalence of metabolic components and syndromes in Northern Ethiopia (n = 1476].

Metabolic syndromes and components	Metabolic syndrome		
	Frequency	Prevalence[95% CI]	Prevalence[95%CI]
		CPR	APR
Large waist circumference	317	21.94[19.85, 24.15]	22.60[19.23–26.00]
Low HDL concentration	1046	70.91[68.47, 73.27]	70.61[67.17, 74.05]
High Tri glyceride concentration	842	57.13[54.60, 59.64]	55.63[51.97, 59.29]
High blood pressure	520	35.21[32.81, 37.67]	39.14[35.46, 42.81]
High fasting blood glucose	297	20.01[18.03, 22.12]	21.72[18.41, 25.03]
At least one metabolic risk factors	1251	90.92[89.27, 92.38]	91.67[90.06, 93.27]
At least two metabolic risk factors	885	64.32[61.72, 66.85]	65.13[61.92, 68.34]
At least three metabolic risk factors	465	33.79[31.29, 36.36]	34.18[30.31, 38.06]
At least four risk factors	174	12.65[10.93, 14.52]	13.63[10.71, 16.55]
All five metabolic risk factors	30	2.18[1.48, 3.10]	3.68[1.32, 6.04]

Note: Abbreviations: CI, Confidence Interval; CPR, Crude Prevalence Rate; APR, Adjusted Prevalence Rate.

### Prevalence of metabolic syndrome

The CPR and APR of MetS were 33.79%; (95%CI: 31.29%–36.36%) and 34.2%; (95% CI: 30.31%–38.06%) respectively. The prevalence of four metabolic syndrome components was two times more than those with five components (Table 2).

### Predictors associated to metabolic syndrome

In the adjusted analysis, currently married (AOR = 1.50; 95%CI: 1.03, 2.19), frequency of alcoholic drink 1–2 day per week (AOR = 0.60; 95%CI: 1.09, 0.71), frequency of alcoholic drink 1–3 days per month (AOR = 0.27; 95%CI:0.1, 0.76), frequency of alcoholic drink less than one per month AOR = 0.28; 95%CI: 0.11, 0.77), eating vegetables four days a week (AOR = 3.69; 95%CI:1.33, 10.22), A salt sauce added in the food sometimes (AOR = 5.06; 95%CI: 5.06 (2.07, 11.34)], heart rate average (AOR = 1.02; 95%CI: 1.01, 1.03), Hemoglobin A1C (AOR = 1.83; 95%CI: 1.49, 2.24), overweight (AOR = 24.28; 95%CI: 10.08, 58.47) and obesity = AOR = 38.81; 95%CI: 12.20, 111.04) showed statistical associations with the MetS (Table 3).

**Table 3:** Logistic regression analysis of factors associated with pre-diabetes and diabetes for study participants in Northern Ethiopia.

Variables	Metabolic syndrome		OR[95%CI]	
	Yes	No	COR	AOR
**Sex**				
Male (Ref)	271	559		NS
Female	194	352	1.14[0.91, 1.43]	
**Marital status**				
Never married (Ref)	79	321		NS
Currently married	339	523	2.63[1.99, 3.49]**	1.50[1.03, 2.19]*
Separated	2	3	2.71[0.45, 16.49]	
Divorced	30	48	2.54[1.51, 4.26]**	
Widowed	15	16	3.30[1.53, 7.15]**	
**Occupation**				
Government employees (Ref)	437	821		NS
Farmer	18	49	0.69[0.40, 1.20]	
Self employed	4	11	0.68[0.22, 2.16]	
Student	1	17	0.11[0.01, 0.83]*	
Home marker	13	15	1.13[0.27, 4.74]	
**Past smoking history**				
Yes (Ref)	26	30		NS
No	436	875	0.57[0.34, 0.98]*	
**Frequency of alcoholic drink**				
Daily (Ref)	24	**5**		NS
5–6 day per week	6	4	0.31[0.06, 1.53]	
1–2 days per week	131	125	0.22[0.08, 0.59]**	0.26[0.09, 0.71]*
1–3 days per month	128	161	0.17[0.06, 0.45]**	0.27[0.1, 0.76]*
Less than one per month	136	351	0.08[0.03, 0.22]**	0.28[0.11, 0.77]*
**Frequency of days eating vegetables per week**				
0 days (Ref)	87	226		
2 days	126	214	1.53[1.10, 2.13]*	
4days	18	16	2.92[1.43, 5.99]**	3.69[1.33, 10.22]*
7days	102	183	1.45[1.02, 2.05]*	
**Frequency of days eating meat**				
Once per month (Ref)	40	117		NS
3–4 times per week	135	227	1.74[1.15, 2.64]**	
5–6 times per week	109	120	2.66[1.71, 4.14]**	
**A salt sauce added in the food**				
Always (Ref)	396	850		
Some times	22	16	2.95[1.53, 5.68]**	5.06[2.07, 12.34]**
Rarely	10	7	3.07[1.16, 8.11]*	
Never	32	30	2.29[1.37, 3.82]**	
**Advised to quite using tobacco**				
Yes (Ref)	17	16		NS
No	440	884	0.47[0.23, 0.94]*	
**Advised to eat fruits per day**				
Yes (Ref)	60	51		NS
No	399	851	0.40[0.27, 0.59]**	
**Advised to reduced fat in the diet**				
Yes (Ref)	61	46		NS
No	399	857	0.35[0.24, 0.52]**	
**Advised to start more physical activity**				
Yes (Ref)	74	59		NS
No	386	844	0.36[0.25, 0.52]**	
**Advised to maintain weight lose**				
Yes (Ref)	51	31		
No	48	871	0.28[0.18, 0.45]**	
**Heart rate average**	**-**	**-**	1.03[1.01, 1.04]**	1.02[1.007, 1.034]*
**Hemoglobin A1C**	**-**	**-**	2.12[1.78, 2.51]**	1.83[1.49, 2.24]**
**BMI**				
Normal (Ref)	218	571		
Under weight	10	187	7.14[3.71, 13.74]**	7.63[3.29, 17.73]**
Overweight	196	136	26.95[13.75, 56.97]**	24.28[10.08, 58.47]**
Obesity	38	15	47.37[19.79, 113.40]**	38.81[12.20, 111.04]**

Note: *p-value<0.05,

** P-value <0.01; Abbreviations: Ref, Reference category; NS, Not statistically significant variable.

## Discussion

The study aimed to assess the magnitude, components and predictors of MetS. It revealed that unadjusted and adjusted prevalence rate were (CPR = 33.79%; 95%CI: 31.29%–36.36%) and (APR = 34.2%; 95% CI: 30.31%–38.06%) respectively. The most prevalent component was low HDL concentration (CPR = 70.91%; 95% CI: 68.47–73.27 and APR = 70.61% 95%CI: 67.17%–74.05%). While, high fasting blood glucose was the least prevalent (CPR = 20.01%; 95%CI: 18.03%–22.12% and APR = 21.72% 95%CI: 18.41%–25.03%). eating vegetables four days a week, a salt sauce added in the food sometimes, overweight, obesity had a strong association with MetS.

Unadjusted and adjusted prevalence rate of metabolic syndrome were (CPR = 33.79%; 95%CI: 31.29%–36.36%) and (APR = 34.2%; 95% CI: 30.31%–38.06%) respectively. Several studies reported the prevalence of MetS worldwide including Africa. These shreds of evidences were quite heterogeneous, which can be attributed to a difference definitions and ways of diagnosis and classification. Hence, this limits direct comparisons with the current study. The adjusted prevalence of the current study (34.2%) lies between finding (12–86%) evidenced from sub Saharan Africa [7]. But, it is higher compared to evidence documented in 10 European countries (24%), the UK (32%) [28]. this variation was due to differences in study settings, socio-cultural, and life style modification among the countries. This prevalence is much lower than a report at Ogbera Lagos, Nigeria (86%) [29], national estimate in Ethiopia (45.9%) [19], Ghana (68.6%) [30], and Iran (64.9%) [31]. These differences could be due to the variation in diagnosis and classification criteria of MetS. The other reason for elevated value of MetS observed in Nigeria and Ghana could be due to the fast urbanization and development. Regarding to residency, the association of MetS and urbanization could be as a result of a sedentary life style, increased intake of calorie rich foods and central obesity. This result is supported by other studies [32, 33]. However, the current finding was consistent with findings from Malaysia (37.4%) [34], Germany (33.7%) [35], Korea (36.1%) [36]. These similarities might be due to the use of the same study design, definition and classification criteria.

The most prevalent component of MetS was low HDL concentration (CPR = 70.91%; 95%CI: 68.47%–73.27%) and (APR = 70.61%; 95%CI: 67.17%–74.05%) followed by high tri-glyceride concentration (CPR = 57.13%; 95%CI: 54.60%–59.64%) and (APR = 55.63%; 95%CI: 51.97%–59.29%). Similar to the current study, low HDL-C was found to be the most prevalent MetS components in black African which close (70.1%) to the current finding [37].

According to the NCEP- ATPIII criteria, where the highest prevalence of MetS was observed, high TG and low HDL were the most frequent abnormal MetS components. Even though there were no previous studies which support or contradict the findings of the current study, there were pieces of evidences that indicated abnormal levels of TG and HDL. This has an implication of adverse health effects in which low level of HDL in the body is associated with an increased risk of CVD, coronary heart diseases and death [38]. Thus, interventions focusing on abnormal TG and HDL need to be prioritized [38, 39]. Besides; a pooled prevalence (44%) of high blood pressure reviewed in Africa [7] was relatively high when compared from the current finding (39.4%). However, close to the continental estimate. The difference might be variation in design and study population in which the later was conducted using a longitudinal study design on DM patients. Even though, comparable evidence was found on WC with the current study, evidence suggested that it had the strongest associations with health risk indicators followed by BMI [40, 41].

Respondents who were currently married were significantly associated with MetS, which is also evidenced from Ethiopia [19], Nigeria [29], and Iran [31]. Eating vegetables in a typical four days a week and a salt sauce added in the food sometimes were 3.7 and 5.1 times more protected from metabolic syndrome compared to their counterparts. This evidence was further supported with studies conducted from Ethiopia [42, 43], and Brazil [44]. Besides, the odds of MetS were 24.3 and 38.8 times higher among respondents with overweight and obesity than the normal. This was almost 8 to 9 times higher when compared with the findings from Ethiopia, Nigeria Ghana, Iran, and Malaysia [19, 29, 30, 31, 34]. The difference might be due to sample size, and study setting variation. Those who drink 1–2 days a week, 1–3 days a month, less than once a month were less likely exposed to MetS than those who drink alcohol daily. Nevertheless, physical activity and use of tobacco were tended to none predictors’ of MetS as similarly reported from Southern Ethiopia [45].

The findings from the present study showed MetS is a major burden. Early identification of visible intervention and awareness creation is a great importance to reduce its occurrence and progression [46].

### Strengths and limitations

The use of digital device for measured biochemical and physical measurements might increase validity and reliability of study findings. Due to the cross—sectional nature of the study the temporal relationship between the outcome and predictor variables might not powerful. The reliance on self-reported data might lead to incorrect estimates. Non-probability convenience sampling was employed and this might have an effect on generalizability.

## Conclusion

The magnitude of MetS was lower than the national estimate but significantly high considering estimations from pocket studies. The predictors were easy to address and targeted interventions of education initiatives, dietary modifications and health screening measures needed to avoid further complications.

## Supporting information

S1 Dataset Dataset used for the analysis of the study. (SAV)

S1 File Data collection tool. (PDF)

## Abbreviations

HDL: high-density lipoprotein
IDF: International Diabetes Federation
MetS: metabolic syndrome
NCEP/ATP: National Cholesterol Education Adult Treatment Panel
WHO: World Health Organization
APR: Adjusted Prevalence Rate
CPR: Crude Prevalence Rate
AOR: adjusted Odds Ratio
UK: United Kingdom
STEPs: STEP wise Approach to Surveillance

## References

[1] GBD 2016 Causes of Death Collaborators. Global, regional, and national age-sex specific mortality for 264 causes of death, 1980–2016: a systematic analysis for the Global Burden of Disease Study 2016. Lancet 2017; 390: 1151–210. doi: 10.1016/S0140-6736(17)32152-9 28919116 PMC5605883

[2] Mathers C. D. and Loncar D., “Projections of global mortality and burden of disease from 2002 to 2030,” PLoS Medicine, vol. 3, no. 11, article e442, 2006. doi: 10.1371/journal.pmed.0030442 17132052 PMC1664601

[3] Kassi E, Pervanidou P, Kaltsas G, Chrousos G. Metabolic syndrome: definitions and controversies. BMC medicine. 2011;9(1). doi: 10.1186/1741-7015-9-48 21542944 PMC3115896

[4] Chawla A, Chawla R, Jaggi S. Microvasular and macro vascular complications in diabetes mellitus: Distinct or continuum? Indian journal of endocrinology and metabolism. 2016; 20(4):546. doi: 10.4103/2230-8210.183480 27366724 PMC4911847

[5] Chang ST, Chu CM, Pan KL, Lin YS, Wang PC, et al. (2011) Prevalence and cardiovascular disease risk differences for erectile dysfunction patients by three metabolic syndrome definitions. Int J Impot Res 23: 87–93. doi: 10.1038/ijir.2011.9 21471983

[6] Pan JJ, Qu HQ, Rentfro A, McCormick JB, Fisher-Hoch SP, et al. (2011) Prevalence of metabolic syndrome and risks of abnormal serum alanine aminotransferase in Hispanics: a population-based study. PLoS One 6: e21515. doi: 10.1371/journal.pone.0021515 21720553 PMC3123360

[7] Okafor CI. The metabolic syndrome in Africa: Current trends. Indian J Endocrinol Metab. 2012;16(1):56–66. doi: 10.4103/2230-8210.91191 22276253 PMC3263198

[8] Ulasi I. I., Ijoma C. K., and Onodugo O. D., “A communitybased study of hypertension and cardio-metabolic syndrome in semi-urban and rural communities in Nigeria,” BMC Health Services Research, vol. 10, article no. 71, 2010. 10.1186/1472-6963-10-71 PMC2858142 20302648

[9] Owolabi E. O., Goon D. T., Adeniyi O. V., Adedokun A. O., and Seekoe E., “Prevalence and correlates of metabolic syndrome among adults attending healthcare facilities in eastern cape, South Africa,” The Open Public Health Journal, vol. 10, pp. 148–159, 2017.

[10] El Brini O., Akhouayri O., Gamal A., Mesfioui A., and Benazzouz B., “Prevalence of metabolic syndrome and its components based on a harmonious definition among adults in Morocco,” Diabetes, Metabolic Syndrome and Obesity: Targets and Therapy, vol. 7, pp. 341–346, 2014. doi: 10.2147/DMSO.S61245 25114576 PMC4124051

[11] Dandji M., Zambou F., Dangang D., Nana F., and Tchouanguep F., “Prevalence of metabolic syndrome in adult men of the dschang health district in Western-Cameroon,” World Journal of Nutrition and Health, vol. 6, no. 1, pp. 1–10, 2018.

[12] Lee CM, Huxley RM, Woodward M, et al. Comparisons of metabolic syndrome definitions in four populations of the Asia-Pacific region. Metab Syndr Relat Disord. 2008; 6(1):37–46. doi: 10.1089/met.2007.0024 18370835

[13] Ford ES. Prevalence of the metabolic syndrome defined by the international diabetes federation among adults in the U.S. Diabetes Care. 2005; 28(11): 2745–9. doi: 10.2337/diacare.28.11.2745 16249550

[14] Ford ES, Giles WH, Dietz WH. Prevalence of the metabolic syndrome among US adults: findings from the third National Health and nutrition examination survey. J Am Med Assoc. 2002; 287(3):356–9. doi: 10.1001/jama.287.3.356 11790215

[15] Motala AA, Mbanya JC, Ramaiya KL. Metabolic syndrome in sub-Saharan Africa. Ethnicity Disease. 2009; 19(2):S2–8. 19537344

[16] Kelliny C, William J, Riesen W, Paccaud F, Bovet P. Metabolic syndrome according to different definitions in a rapidly developing country of the African region. Cardiovasc Diabetol. 2008; 7: article no. 27. 10.1186/1475-2840-7-27 PMC2556312 18801172

[17] Mbugua SM, Kimani ST, Munyoki G. Metabolic syndrome and its components among university students in Kenya. BMC Public Health. 2017;17(1):909. Published 2017 Nov 28. doi: 10.1186/s12889-017-4936-x 29183300 PMC5704380

[18] Solomon S., Mulugeta W. Disease burden and associated risk factors for metabolic syndrome among adults in Ethiopia. BMC Cardiovasc Disord 19, 236 (2019). 10.1186/s12872-019-1201-5 31655560 PMC6815352

[19] Tran A, Gelaye B, Girma B, Lemma S, Berhane Y, Bekele T, et al. Prevalence of metabolic syndrome among working adults in Ethiopia. Int J Hypertens. 2011; 2011:193719. doi: 10.4061/2011/193719 21747973 PMC3124293

[20] Grundy S. M., Cleeman J. I., Daniels S. R. et al., “Diagnosis and management of the metabolic syndrome: an American Heart Association/National Heart, Lung, and Blood Institute scientific statement,” Circulation, vol. 112, no. 17, pp. 2735–2752, 2005. doi: 10.1161/CIRCULATIONAHA.105.169404 16157765

[21] Alberti K. G. M. M., Zimmet P., and Shaw J., “The metabolic syndrome—a new worldwide definition,” Lancet, vol. 366, no. 9491, pp. 1059–1062, 2005. doi: 10.1016/S0140-6736(05)67402-8 16182882

[22] Gebremariam LW, Chiang C, Yatsuya H, et al. Non-communicable disease risk factor profile among public employees in a regional city in northern Ethiopia. Sci Rep. 2018; 8(1):9298. Published 2018 Jun 18. doi: 10.1038/s41598-018-27519-6 29915239 PMC6006379

[23] NCDs/STEPS manual- World Health Organization; www.who.int/ncds/surveillance/steps/manual/en/.

[24] Salt reduction, fact sheetN0393. Geneva: World Health Organization;2015

[25] World Health Organization. Global physical activity questionnaire: analysis guide, http://www.who.int/ncds/surveillance/steps/resources/GPAQ_Analysis_Guide.pdf (Accessed 1 April 2018).

[26] World Health Organization. Healthy diet, http://www.who.int/mediacentre/factsheets/fs394/en/ (Accessed 6 April 2018).

[27] National Institute on Alcohol Abuse and Alcoholism. Drinking levels defned, https://www.niaaa.nih.gov/alcohol-health/overviewalcohol-consumption/moderate-binge-drinking (Accessed 1 April 2018).

[28] Scuteri A, Laurent S, Cucca F, Cockcroft J, Cunha PG, Mañas LR, et al. Metabolic syndrome across Europe: different clusters of risk factors. Eur J Prev Cardiol. 2015;22(4):486–91. doi: 10.1177/2047487314525529 24647805 PMC4544872

[29] Ogbera AO. Prevalence and gender distribution of the metabolic syndrome. Diabetol Metab Syndr. 2010; 2:1. doi: 10.1186/1758-5996-2-1 20180954 PMC2836983

[30] Abagre TA. Metabolic syndrome among people with type 2 diabetes mellitus in two selected hospitals in the Brong Ahafo Region. Doctoral dissertation, University of Ghana; 2016.

[31] Foroozanfar Z, Najafpour H, Khanjani N, Bahrampour A, Ebrahimi H. The prevalence of metabolic syndrome according to different criteria and its associated factors in type 2 diabetic patients in Kerman, Iran. Iran J Med Sci. 2015; 40(6):522. 26538781 PMC4628143

[32] Bouguerra R, Ben Selam L, Alberti H, Ben Rayana C, El Atti J, Blouza S, et al. Prevalence of metabolic abnormalities in the Tunisian adults: a population based study. Diabete Metab. 2006; 32(3):215–21. doi: 10.1016/s1262-3636(07)70271-9 16799397

[33] Chowdhury MZI, Anik AM, Farhana Z, et al. Prevalence of metabolic syndrome in Bangladesh: a systematic review and meta-analysis of the studies. BMC Public Health. 2018; 18:308. doi: 10.1186/s12889-018-5209-z 29499672 PMC5833131

[34] Ramli AS, Daher AM, Nor-Ashikin MNK, Mat-Nasir N, Ng KK, Miskan M, et al. JIS defnition identifed more Malaysian adults with metabolic syndrome compared to the NCEP-ATP III and IDF criteria. Biomed Res Int. 2013; 2013:1–10. 10.1155/2013/760963 PMC3794630 24175300

[35] Van Vliet-Ostaptchouk J.V., Nuotio M., Slagter S.N. et al. The prevalence of metabolic syndrome and metabolically healthy obesity in Europe: a collaborative analysis of ten large cohort studies. BMC Endocr Disord 14, 9 (2014). 10.1186/1472-6823-14-9 24484869 PMC3923238

[36] Oh J, Hong Y, Sung Y-A, Barrett-Connor E: Prevalence and Factor Analysis of Metabolic Syndrome in an Urban Korean Population. Diabetes care 2004, 27(8):2027–2032. doi: 10.2337/diacare.27.8.2027 15277435

[37] Crowther NJ, Norris SA. The current waist circumference cut point used for the diagnosis of metabolic syndrome in sub-Saharan African women is not appropriate. PLoS One. 2012; 7:e48883. doi: 10.1371/journal.pone.0048883 23145009 PMC3493601

[38] Callaghan BC, Feldman E, Liu J, et al. Triglycerides and amputation risk in patients with diabetes: ten-year follow-up in the DISTANCE study. Diabetes Care. 2011;34(3):635–40. 10.2337/dc10-0878 21285390 PMC3041196

[39] Ahmed HM, Miller M, Nasir K, McEvoy JW, Herrington D, Blumenthal RS, et al. Primary low level of high-density lipoprotein cholesterol and risks of coronary heart disease, cardiovascular disease, and death: results from the multi-ethnic study of atherosclerosis. Am J Epidemiol. 2016; 183(10):875–83. doi: 10.1093/aje/kwv305 27189327 PMC4867155

[40] DeFina LF, Vega GL, Leonard D, Grundy SM. Fasting glucose, obesity, and metabolic syndrome as predictors of type 2 diabetes: the Cooper Center Longitudinal Study. J Investig Med. 2012; 60(8):1164–1168. doi: 10.2310/JIM.0b013e318275656a 23111652

[41] Shen W, Punyanitya M, Chen J, et al. Waist circumference correlates with metabolic syndrome indicators better than percentage fat. Obesity (Silver Spring). 2006; 14(4):727–736. doi: 10.1038/oby.2006.83 16741276 PMC1894647

[42] Gebremeskel G.G., Berhe K.K., Belay D.S. et al. Magnitude of metabolic syndrome and its associated factors among patients with type 2 diabetes mellitus in Ayder Comprehensive Specialized Hospital, Tigray, Ethiopia: a cross sectional study. BMC Res Notes 12, 603 (2019) 10.1186/s13104-019-4609-1 31533851 PMC6751785

[43] MOH. The Federal Democratic Republic of Ethiopia Ministry of Health: health sector transformation plan. 2015/2016.

[44] De Oliviera EP, McLellan KCP, de Silveira LVA, Burini RC. Dietary factors associated with metabolic syndrome in Brazilian adults. Nutrition. 2012; 11:13. 10.1186/1475-2891-11-13 PMC3337297 22417631

[45] Tesfaye DY, Kinde S, Medhin G, Megerssa YC, Tadewos A, Tadesse E, et al. Burden of metabolic syndrome among HIV-infected patients in Southern Ethiopia. Diabetes Metab Syndr Clin Res Rev. 2014; 8:102–7. doi: 10.1016/j.dsx.2014.04.008 24907175

[46] Pitsavos C, Panagiotakos D, Weinem M, Stefanadis C. Diet, exercise and the metabolic syndrome. Rev Diabet Stud. 2006; 3(3):118–26. doi: 10.1900/RDS.2006.3.118 17487335 PMC1783583

