# Supplementary material for: Magnitude, components and predictors of metabolic syndrome in Northern Ethiopia: Evidences from regional NCDs STEPS survey, 2016
Source: PLoS One. 2021 Jun 21;16(6):e0253317. doi: 10.1371/journal.pone.0253317 (PMC8216523; doi:10.1371/journal.pone.0253317)
Supplement: Attachment — Submitted filename: Response to Reviewers..docx [file pone.0253317.s003.docx]

**Responses to Reviewer’s and editor comments**

**Journal name:** PLOS ONE

**Corresponding Author:** Kiros Fenta Ajemu

We would like to express our appreciation to the reviewer for their constructive and supportive comments in the first version of the manuscript entitled ***“Magnitude, components and predictors of metabolic syndrome in Northern Ethiopia: Evidences from regional NCDs STEPS survey, 2016”****.* This will be an input to improve the quality of the manuscript. We have meticulously revised the manuscript and incorporated all the changes in the revised version of the manuscript based on the suggestions and comments made by the reviewer. We have highlighted these changes in the manuscript. We have listed all the responses for each of the comment/ suggestion made by the reviewer as follows:

**Responses to comments Academic Editor: Mr. Paolo Magni**

**Comment 1:** Please ensure that your manuscript meets PLOS ONE's style requirements, including those for file naming.

**Response 1:** Thank you for your constructive comment. We crosscheck the manuscript based on the PLOS ONE style requirements in line with the web site you suggested.

**Comment 2:** We noted that “**Figure 1**” in your submission contains map images which may be copyrighted.

**Response 2:** Thank you for your transparent and constructive comment. Actually we made the map using regional GPS data but as you have said the map is not that much relevant since Mekelle city is already known and everybody can Google it from internet. Therefore we agreed to remove the figure “**Figure 1**” from the main body of the manuscript. The change made is listed below as per your comments and suggestions in:

- **Material and Methods section Line (70)**, **Page (4)**].

**Responses to reviewer comments**

**Comment 1:** The title of the article is "Magnitude, components and predictors of metabolic syndrome in Northern Ethiopia: Evidences from regional NCDs STEPS survey, 2016".The authors conducted a community based cross sectional study. This study aimed to assess magnitude, components, and predictors of metabolic syndrome in Tigray region northern Ethiopia, 2016.This is a quite interesting study.

**Response 1:** thank you for your acknowledgment that makes me to do more.

**Comment 2:** in the statistical analyses, please clarify what method that used for adjusting in multivariate analysis? Please provide test for interaction between variables, goodness of fit, and multicollinearity.

**Response 2:** thank you for your consideration in statical analysis in line with data handling mechanisms since it is the back bone and pillar to find the real evidence and minimize biases and errors. The changes that we made were listed below as per your comments and suggestions in **Method section**, **Line (137-144)**, **Pages (7)**].

- **Test of interaction, Line (142-143)**, **Pages (7)**].
- **Goodness of fit, Line (140-142)**, **Pages (7)**].
- **Multicollinearity, Line (145-146)**, **Pages (7)**].

**Comment 3:** If non-probability convenience sampling was employed. This limitation of the study might affect generalizability.

**Response 3:** thank you and I accept you fear of generalizability but we put it as a limitation in the:

- **Strength and Limitation section, Line (243-244), pages (15)]. As part of the limitation of the study**

**Comment 4:** Who interviewed the participants? Were they doctors, nurses, medical students, or research investigators? Were they trained before administered the questionnaires, by how many people, in how long time, where? The time of interview for each person?

**Response 4:** thank you your concern for data quality and the changes made were explained in **Method section**, **Line (94- 103**] **Page (5)]**

- **Who interviewed, Line (96-97)**, **Pages (5)**].
- **How many people, Line (96-97)**, **Pages (5)**].
- **How long and where, Line (102-103)**, **Pages (5)**].

**Comment 5:** The main concern is that the questionnaires should be validated and have good reliability and validity. Reliability of questionnaires should be mentioned Please provide citation and reference of the questionnaire.

**Response 5:** thank you for your comment and the changes made were listed in Method section **Line (94-95**] **Page (5)]**

- **Validated questionnaire, Line (94)**, **Pages (5)**].
- **Reference, Line (95)**, **Pages (5)**].

**Comment 6:** Please describe the detail of Tigray region northern Ethiopia; such as is it rural or urban community, the number of population and population structure.
**Response 6:** thank you for your concern in the study area and the changes made were listed in Method section **Line (70-77**] **Page (4)]**

**Comment 7:** It is important that within the manuscript, the authors clarify the importance of this work, how it differs from and advances previously published work and how this article can benefit the field and patients in the future etc.

**Response 7:** the previously published work has different objective and it was a scientific report on prevalence of behavioral characteristics such as alcohol use, eating diet, physical activity, diabetes and pre-diabetes prevalence. The study did not address predictor factors the magnitude of association in respective cardiovascular disease. The current study will provide detail of the risk factors for future deployment of visible intervention. For further information you can access the article on line Journal of scientific report <https://www.ncbi.nlm.nih.gov/pmc/articles/PMC6006379/>.

***Once again thank you for your strong and committed concern for improving the manuscript***

***Sincerely your’s*** Kiros Fenta Ajemu (*kirosfenta@gmail.com)*Researcher, Tigray Health Research Institute, Mekelle, Tigray, Ethiopia
